# Mechanism of Lethal Skin Toxicities Induced by Epidermal Growth Factor Receptor Inhibitors and Related Treatment Strategies

**DOI:** 10.3389/fonc.2022.804212

**Published:** 2022-02-10

**Authors:** Yanping Li, Ruoqiu Fu, Tingting Jiang, Dongyu Duan, Yuanlin Wu, Chen Li, Ziwei Li, Rui Ni, Li Li, Yao Liu

**Affiliations:** Department of Pharmacy, Daping Hospital, Army Medical University, Chongqing, China

**Keywords:** EGFR inhibitors, lethal skin toxicities, drug induced disease, treatment strategies, preventive measures

## Abstract

Epidermal growth factor receptor (EGFR) inhibitors are widely used to treat various types of cancers such as non-small cell lung cancer, head and neck cancer, breast cancer, pancreatic cancer. Adverse reactions such as skin toxicity, interstitial lung disease, hepatotoxicity, ocular toxicity, hypomagnesemia, stomatitis, and diarrhea may occur during treatment. Because the EGFR signaling pathway is important for maintaining normal physiological skin function. Adverse skin reactions occurred in up to 90% of cancer patients treated with EGFR inhibitors, including common skin toxicities (such as papulopustular exanthemas, paronychia, hair changes) and rare fatal skin toxicities (e.g., Stevens–Johnson syndrome, toxic epidermal necrolysis, acute generalized exanthematous pustulosis). This has led to the dose reduction or discontinuation of EGFR inhibitors in the treatment of cancer. Recently, progress has been made about research on the skin toxicity of EGFR inhibitors. Here, we summarize the mechanism of skin toxicity caused by EGFR inhibitors, measures to prevent severe fatal skin toxicity, and provide reference for medical staff how to give care and treatment after adverse skin reactions.

## Introduction

The epidermal growth factor receptor (EGFR, also named HER1) is a 170 kDa transmembrane glycoprotein receptor that is coded by the c-erbB1 proto-oncogene located on the human 7q22 chromosome (1). Asparagine-linked glycosylation is a post-translational modification necessary for its active function (2). EGFR is a member of the ErbB receptor family of tyrosine protein kinases, which also includes ErbB-2 (HER2), ErbB-3 (HER3), and ErbB-4 (HER4) (3). EGFR is highly expressed in lung cancer (4), breast cancer, human glioblastoma (5), gastric carcinoma (3), rectal cancer, and head and neck cancer (6) compared to healthy tissues. The EGFR signaling pathway is involved in normal biological processes of cells, and the destruction of the dynamic balance will lead to pathological changes in healthy tissues. Overexpression of EGFR promotes cell proliferation, adhesion, metastasis, and angiogenesis and inhibits apoptosis, all of which can induce tumorigenesis (7). Therefore, EGFR inhibitors have been utilized for cancer treatment.

EGFR inhibitors are divided into monoclonal antibodies (mAb) and small molecule intracellular tyrosine kinase inhibitors (TKIs). EGFR mAb competitively inhibit ligand binding to EGFR extracellular domain with higher affinity than ligand to reduce EGFR signaling pathway activity (8). The small molecule EGFR-TKIs are ATP analogs that competitively bind to the intracellular catalytic domain of EGFR, which blocks ATP-mediated phosphorylation (9). Although EGFR inhibitors have good efficacy for a variety of tumors, adverse reactions such as skin toxicity, interstitial lung disease, hepatotoxicity, ocular toxicity, hypomagnesemia, stomatitis, and diarrhea may occur during treatment (10). These adverse reactions lead to organ, tissue, and system damage, resulting in corresponding drug induced diseases. Finally reduce patient compliance and even lead to the withdrawal of antitumor drugs. Skin toxicities is one of the most common adverse reactions caused by EGFR inhibitors

These skin toxicities may result in fatal complications if they are ignored (11). Doctors, pharmacists, and nurses must consider how to avoid severe skin toxicity in their patients and determine which patients would be prone to fatal skin toxicity. Understanding the molecular and cellular mechanism of skin toxicity and the relationship between skin toxicity and drug efficacy is essential for safe, effective, and rational use of EGFR inhibitors. Here, we focus on the mechanism of skin toxicity and fatal skin toxicity caused by EGFR inhibitors and clinical countermeasures as a mean to alleviate adverse reactions and ultimately achieve the purpose of reducing adverse emotions of the patients during the treatment phase, improving medication compliance, and effectively treating related cancers.

## Activation Mechanism of EGFR Signaling Pathway

**Figure 1:**
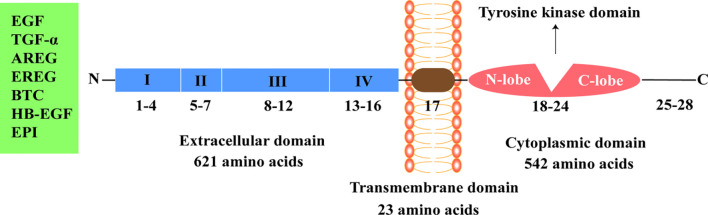
Epidermal growth factor receptor structure. EGF, epidermal growth factor; TGF-α, transforming growth factor-α; AREG, amphiregulin; EREG, epiregulin; BTC, betacellulin; HB-EGF, heparin binding epidermal growth factor-like growth factor; EPI, epiregulin.

The four members of the human ErbB family have similar structures that are divided into the extracellular domain, transmembrane domain, cytoplasmic domain, and C-terminal tail domain (3). Mature EGFR consists of 1186 amino acid residues and is divided into three parts from N-terminal to C-terminal: extracellular domain (621 amino acids), hydrophobic lipophilic short transmembrane domain (23 amino acids), and cytoplasmic domain (542 amino acids) (**Figure 1**).

The extracellular region of EGFR can be subdivided into four domains: I, II, III, and IV. Domains I (amino acids 1-133, exons 1-4) and III (amino acids 313-445, exons 8-12) are rich in leucine and are the main fragments involved in ligand binding in the extracellular domain (7). Domains II (amino acids 134-312, exons 5-7) and IV (amino acids 446-621, exons 13-16) contain 51 cysteine residues and are not involved in ligand binding. However, domain II is involved in the formation of homodimers and heterodimers with other members of the ErbB family (7, 12, 13).

The specific ligands of EGFR include epidermal growth factor (EGF), transforming growth factor-α, and amphiregulin, while non-specific ligands include epiregulin, betacellulin, heparin binding EGF-like growth factor, and epiregulin (14, 15). EGF, the ligand of EGFR, was first isolated from the mouse submandibular gland and is associated with epidermal proliferation and keratinization (16). Asparagine-linked glycosylation is a post-translational modification necessary for functional EGFR, and the extracellular domain of EGFR contains 12 sites for asparagine-linked glycosylation (2, 12). The transmembrane domain of EGFR (amino acids 622-644, exon 17) serves to link the two functional domains of the extracellular and cytoplasmic domains (13). The cytoplasmic domain of EGFR (amino acids 645-1186, exons 18-28) includes a tyrosine kinase domain (exons 18-24) and C-terminal tail (exons 25-28). The tyrosine kinase domain can be subdivided into the N-lobe and C-lobe. ATP binds to the gap formed by the two lobes. EGFR-TKIs inhibit the activation of tyrosine kinase and subsequent signaling pathways by competitively binding the ATP-binding site of the tyrosine kinase domain (17–19).

**Figure 2:**
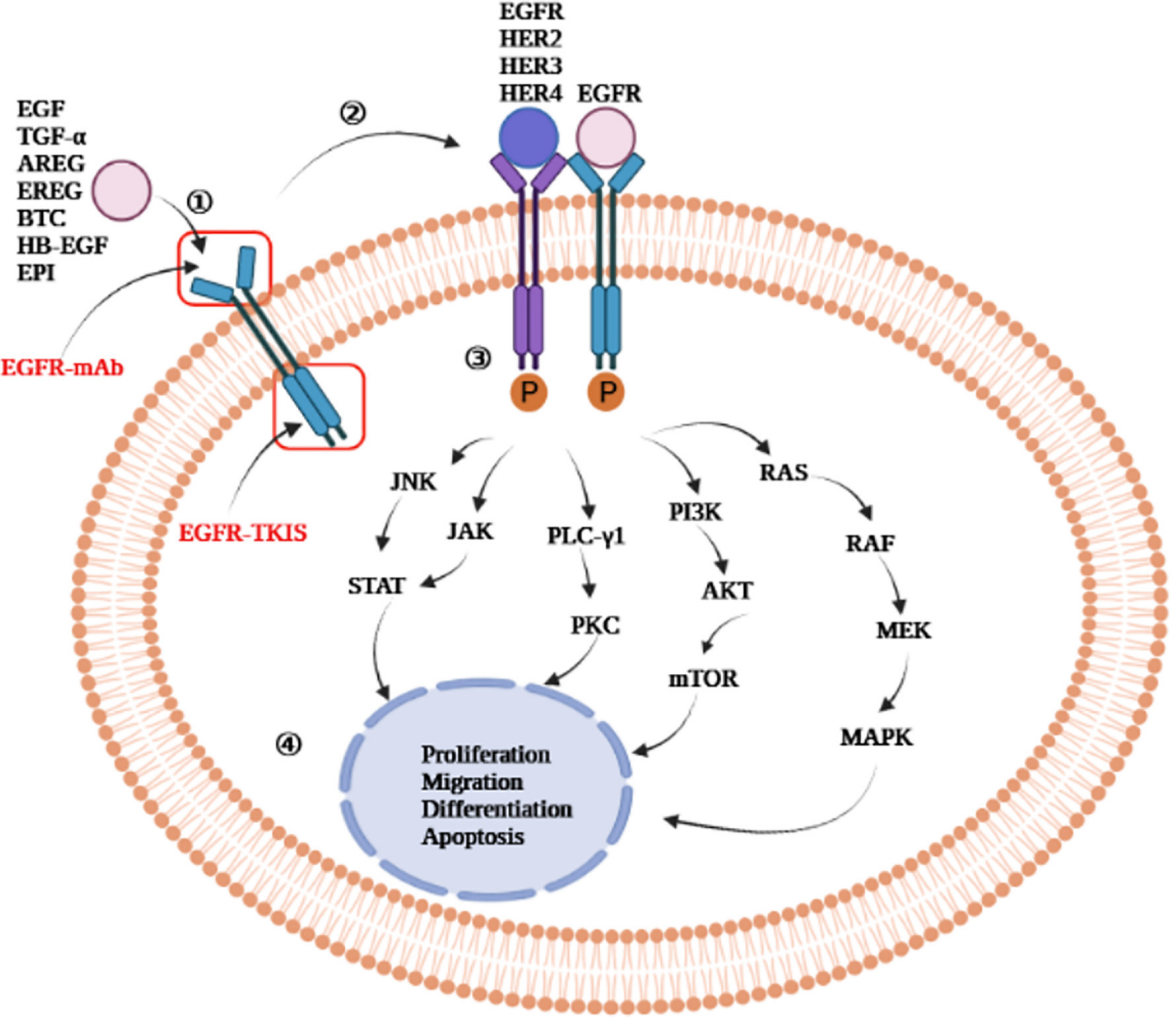
Epidermal growth factor receptor activation mechanism. EGFR-mAb: Cetuximab, panitumumab, zalutumumab, nimotuzumab; EGFR-TKIs: gefitinib, erlotinib, lapatinib, icotinib, neratinib, dacomitinib, afatinib, olmutinib, osimertinib, furmonertinib mesylate, brigatinib; JNK, jun amino-terminal kinase; JAK, janus activated kinase; STAT, signal transducer and activator of transcription; PLC-γ1, phospholipase C-γ1; PKC, protein kinase C; MAPK, mitogen-activated protein kinase; PI3K, phosphatidylinositol-3- kinase; mTOR, mammalian target of rapamycin; AKT, protein kinase B; RAS, rat sarcoma virus gene homolog; RAF, rapidly accelerated fibrosarcoma serine/threonine kinase; MEK, mitogen-activated protein kinase kinase; ERK, extracellular signal-regulated kinase; EGF, epidermal growth factor; TGF-α, transforming growth factor-α; AREG, amphiregulin; EREG, epiregulin; BTC, betacellulin; HB-EGF, heparin binding epidermal growth factor-like growth factor; EPI, epiregulin; EGFR, epidermal growth factor receptor; HER, human epidermal growth factor receptor; mAb, monoclonal antibody; TKIs, tyrosine kinase inhibitors.

EGFR is activated in four phases (7, 20–22) (**Figure 2**): 1) The ligand binds to the extracellular domain of EGFR; 2) Homodimerization or heterodimerization with ErbB-2, ErbB-3, and ErbB-4 (also known as HER-2, HER-3, and HER-4, respectively) occurs. ErbB-2 is the most common heterodimerization partners of EGFR; 3) Autophosphorylation of tyrosine residues in the cytoplasmic domain occurs; 4) The activation of the intracellular signaling pathway occurs, which regulates cell proliferation, migration, differentiation, and apoptosis.

## EGFR Inhibitors Used Clinically

**Figure 3:**
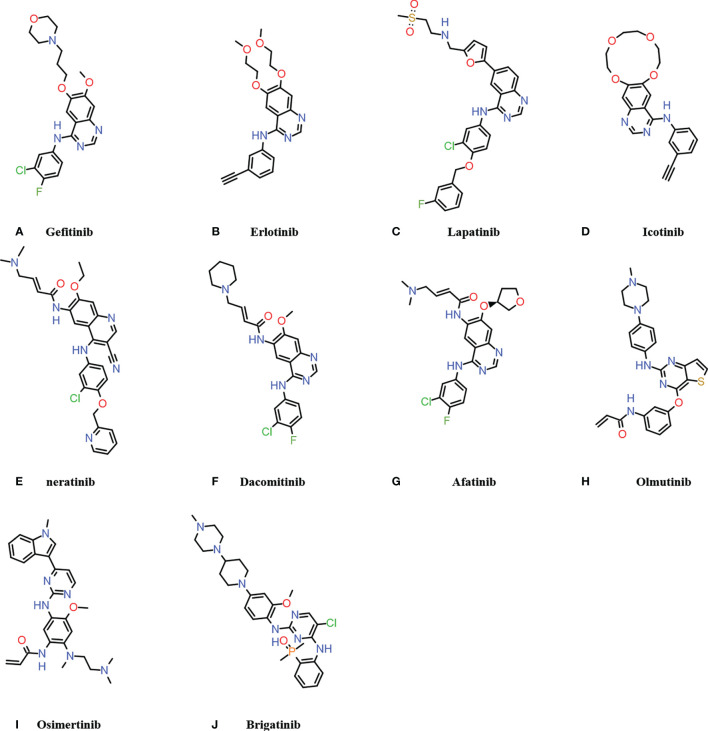
Chemical formula of clinically used EGFR-TKIs. **(A–D)** are the first generation EGFR-TKIs; **(E–G)** are the second generation EGFR-TKIs; **(H, I)** are the third generation EGFR-TKIs; **(J)** is the fourth generation EGFR-TKIs.

At present, there are four generations of EGFR-TKIs (**Table 1**) and multiple EGFR-mAbs (**Table 2**) that have been developed, such as cetuximab, panitumumab, zalutumumab, and nimotuzumab. The chemical formula of EGFR-TKIs for clinical use is shown in **Figure 3**.

**Table 1:** Clinically used EGFR-TKIs.

Classification	Molecular mechanism	Drug name	Targets	Clinical application	Severe skin toxicity (reference)
First generation EGFR-TKIs	The first generation EGFR-TKIs can reversibly inhibit EGFR phosphorylation by competitive binding of tyrosine kinase catalytic structure with ATP through noncovalent bonds (23)	Gefitinib (ZD1839)	EGFR	NSCLC (24)	AGEP (25); TEN (26, 27); NME (28)
		Erlotinib (CP-358774)	EGFR; EGFR (del19); EGFR (L858R)	NSCLC; Pancreatic cancer (29)	SJS (30); TEN (31); AGEP (32)
		Lapatinib (GW572016)	EGFR; HER2	Breast cancer (33)	AGEP (34)
		Icotinib (BPI-2009)	EGFR (T790M); EGFR (L858R); EGFR (L861Q)	NSCLC (35)	DIHS (35)
Second generation EGFR-TKIs	The second generation of EGFR-TKIs irreversibly inhibits multiple ErbB receptors by competitively binding to the tyrosine kinase catalytic domain with ATP *via* a covalent bond (36)	Neratinib (HKI-272)	EGFR; HER2; HER4	Breast cancer (37)	
		Dacomitinib (PF-00299804)	EGFR; EGFR (del19); EGFR (L858R); HER2; HER4 (38);	NSCLC (24)	
		Afatinib (BIBW 2992)	EGFR; EGFR (L858R); HER2; HER4	NSCLC (39)	SJS (40); DIHS (41); SJS/TEN (42); SJS (39)
Third generation EGFR-TKIs	The third generation of EGFR-TKIs covalently binds to the ATP-binding site, CYS797, of the EGFR tyrosine kinase domain (43, 44)	Olmutinib (HM61713/BI1482694)	EGFR; EGFR (del19); EGFR (L858R); EGFR (T790M)	NSCLC (45)	SJS/TEN (45)
		Osimertinib (AZD9291)	EGFR; EGFR (del19); EGFR (L858R); EGFR (T790M)	NSCLC (46)	SJS (47); TEN (48)
		Furmonertinib mesylate (AST2818)	EGFR (del19); EGFR (L858R); EGFR (T790M)	NSCLC (49)	
Fourth generation EGFR-TKIs	The fourth generation of EGFR-TKIs irreversibly binds to the ATP binding pocket of C797S/T790M/activating mutation (triple mutation) of EGFR (50)	Brigatinib (AP26113)	EGFR; ALK; ROS1; IGF-1R; FLT-3	ALK-positive NSCLC (51)	

Del19, exon 19 deletion; L858R, exon 21 mutations; T790M, mutation of the 790th amino acid threonine of EGFR to methionine; C797S, cysteine is replaced by serine at position 797; EGFR, epidermal growth factor receptor; TKIs, tyrosine kinase inhibitors; NSCLC, non-small cell lung cancer; AGEP, acute generalized exanthematous pustulosis; TEN, toxic epidermal necrolysis; NME, necrolytic migratory erythema; SJS, Stevens-Johnson syndrome; HER, human epidermal growth factor receptor; DIHS, drug-induced hypersensitivity syndrome; ALK, anaplastic lymphoma kinase; IGF-1R, insulin-like growth factor-1 receptor; FLT-3, fms-like tyrosine kinase 3; Ig, immunoglobulin; mCRC, metastatic colorectal cancer.

**Table 2:** Clinically used EGFR-mAbs.

Classification	Molecular mechanism	Drug name	Targets	Clinical application	Severe skin toxicity (reference)
EGFR-mAb	Cetuximab is a chimeric IgG1 mAb that competes with endogenous ligands to bind to the extracellular domain of EGFR (52)	Cetuximab (IMC-C225)	EGFR	Head and neck cancer (52); mCRC (53)	SJS (25) TEN (54)
	Panitumumab is a fully human IgG2 mAb that competitively inhibits endogenous ligand binding to the extracellular domain of EGFR (55)	Panitumumab (ABX-EGF)	EGFR	mCRC (55)	SJS (56)
	Zalutumumab is a fully human IgG1 mAb that targets the ligand-binding extracellular domain III of EGFR (57)	Zalutumumab (HuMax-EGFr)	EGFR	HNSCC (58)	
	Nimotuzumab is a humanized IgG1 mAb that competitively binds to the extracellular domain III (amino acids 353-358) of EGFR with ligands (59, 60)	Nimotuzumab (h-R3)	EGFR	HNSCC (53) NPC (61)	

EGFR, epidermal growth factor receptor; mAb, monoclonal antibody; SJS, stevens-Johnson syndrome; TEN, toxic epidermal necrolysis; Ig, immunoglobulin; mCRC, metastatic colorectal cancer; HNSCC, head and neck squamous cell carcinoma; NPC, nasopharyngeal carcinoma.

The first-generation EGFR-TKIs includes gefitinib, erlotinib, lapatinib, and icotinib. Gefitinib was the first agent designed to receive approval from the United States Food and Drug Administration (FDA) for the treatment of lung cancer (62, 63). Gefitinib was considered to be safe and effective for adjuvant treatment of operable stage II-IIIA non-small cell lung cancer (NSCLC) in addition to the treatment of conventional mutated NSCLC (64, 65). Seong et al. (28) reported a rare case of necrolytic migratory erythema during the use of gefitinib. Gefitinib has also been reported to cause fatal skin toxicity such as toxic epidermal necrolysis (TEN) (26) and acute generalized exanthematous pustulosis (AGEP) (25). Although gefitinib shows excellent antitumor effects, it was eventually discontinued due to these severe skin toxicities. Erlotinib, a derivative of quinazoline was approved by the FDA on November 18, 2004 for use in NSCLC with exon 19 deletion (del19) or exon 21 point mutation (L858R) (66). Erlotinib often causes papulopustular exanthemas characterized by pruritus (67). Lapatinib (GW572016) is an ATP-competitive, reversible small-molecule inhibitor of ErbB-2 and EGFR tyrosine kinases that has been approved for the treatment of patients with metastatic breast cancer (68–71).

The mutation of amino acid 790 from threonine to methionine (T790M) increases the affinity of EGFR for ATP, which competitively reduces the efficacy of EGFR-TKIs. Therefore, EGFR (T790M) is one of the reasons for resistance to first-generation EGFR-TKIs (68, 72). In order to overcome drug resistance, the second-generation EGFR-TKIs, afatinib and dacomitinib, were developed (73). Dacomitinib was approved by the FDA on September 27, 2018 for the treatment of NSCLC patients with EGFR del19 or exon 21 L858R mutations (73). However, second-generation EGFR-TKIs were not able to be administered at full strength to inhibit T790M mutant lung cancer due to adverse side effects, such as rash caused by inhibition of normal cells (23). Ding et al. (74) concluded in a meta-analysis of clinical trials that afatinib resulted in a higher risk of rash than erlotinib or gefitinib.

In order to overcome the resistance of first and second-generation EGFR-TKIs, the third-generation EGFR-TKIs were developed. Osimertinib was approved by the FDA in 2015 to treat NSCLC patients with the EGFR T790M mutation (75, 76). In March 2021, furmonertinib mesylate was first approved in China for the treatment NSCLC patients with the EGFR T790M mutation (49). Osimertinib is no longer the only third generation EGFR-TKIs approved for the treatment of EGFR T790M mutant NSCLC. However, during the application of the third-generation EGFR-TKIs, a cysteine-to-serine mutation (C797S) occurred at C797 in the kinase binding site. The C797S mutation blocks the formation of a covalent bond at 797, which ultimately reduced the efficacy of the third-generation EGFR-TKIs (77, 78).

EGFR C797S is the most common tertiary mutation in patients with T790M-positive NSCLC treated with third-generation EGFR-TKI osimertinib. In order to overcome the EGFR C797S mutation, brigatinib was developed as a fourth-generation EGFR-TKI. It is effective against the EGFR C797S-T790M-del19 triple mutant (79). Brigatinib received approval for the treatment of anaplastic lymphoma kinase-positive metastatic NSCLC patients who had progressive disease while taking crizotinib or who were intolerant to crizotinib (80).

## Cellular and Molecular Mechanism of Skin Toxicity Caused by EGFR Inhibitors

Skin is the first line of defense against the invasion of external pathogens. Skin structure from the outside to the inside is the epidermis, dermis, and subcutaneous tissue. The skin contains accessory organs such as nails, sebaceous glands, sweat glands, hair follicles, cutaneous nerves, and subcutaneous blood vessels. EGFR is widely expressed in skin keratinocytes, dendritic cells, connective tissue cells, and skin appendage organelles (e.g. sebaceous glands, sweat glands, and hair follicles) and associated with proliferation, apoptosis, migration, and differentiation of normal cells (81–84). Normal activation of EGFR signaling promotes wound healing, inhibits inflammation, and stimulates capillary constriction (85).

EGFR is widely distributed in the skin, and skin toxicity is one of the most common adverse reactions for EGFR inhibitor treatment. EGFR-mAbs generally produce more severe skin toxicity than EGFR-TKIs (14). Rare purpuric drug eruptions have been reported when using EGFR-TKIs such as gefitinib, erlotinib and afatinib. The main clinical manifestations are purpuric macules, papules, and confluent plaques on the lower extremities. These adverse side effects occur because blocking EGFR leads to endothelial inflammation, decreased vascular tone, and ultimately increased vascular permeability (86). Besides the common rash, rare severe lethal skin toxicities from EGFR inhibitors, such as Stevens-Johnson syndrome (SJS), TEN, and AGEP, are often important causes of drug discontinuation. SJS (10% mortality) and TEN (50% mortality) are two related skin and mucosal diseases caused by delayed drug hypersensitivity. They are characterized by extensive epidermal necrosis and skin detachment (the range of detached surface area:SJS < 10%, TEN > 30%, and SJS/TEN = 10%-30%) (87, 88). AGEP is characterized by the formation of sterile non-follicular pustules on the base of the erythema, often accompanied by neutrophilia and fever, which can involve multiple organs in severe cases and may be life-threatening in approximately 4% of patients (25, 89).

Because EGFR homodimers are typically associated with normal skin tissue and primary keratinocytes (90), it is speculated that EGFR inhibitors block activation of EGFR due to the inability of EGFR to homodimerize. Therefore, skin toxicity in normal cells occurs. However, the pathophysiology and mechanisms of skin toxicity caused by EGFR inhibitors have not been fully elucidated. We explain the causes of skin toxicity caused by EGFR inhibitors from the following four aspects: destruction of the physical barrier of the skin by damage to the epidermal layer, damage of hair follicles, destruction of skin homeostasis, inflammation, and host immune activation, and radiotherapy.

### Destruction of the Physical Barrier of the Skin by Damage to the Epidermal Layer

Keratinocytes stratify into enucleated flattened surface squames to form a skin barrier that moisturizes and isolates pathogens. The barrier is maintained by the precise proliferation and differentiation of keratinocytes (91, 92). EGF promotes keratinocyte proliferation by increasing Ki67 and filaggrin expression through the rapidly accelerated fibrosarcoma serine/threonine kinase/mitogen-activated protein kinase kinase/extracellular signal-regulated kinase signaling pathway (93). In addition, EGFR regulates the terminal differentiation of keratinocytes through the phospholipase C-γ1-protein kinase C pathway to maintain and continuously regenerate the epidermal barrier (94). EGFR inhibitors can lead to destruction of physical and immune balance barriers in the epidermis, which results in skin toxicity such as dryness and rashes (93). Claudins, as essential components for the formation of tight junctions, are critical for maintaining the normal skin barrier (95). Fang et al. (96) found that gefitinib may damage the skin barrier by reducing claudin-1 and claudin-4 and increasing claudin-2 expression in keratinocytes, resulting in skin toxicity.

The epidermis is composed of five parts: basal layer, spinous layer, granular layer, stratum lucidum, and stratum corneum. EGFR is abundant in keratinocytes in the basal layer of the epidermis (97). Upon separation of proliferating basal keratinocytes from the basement membrane, they cross the spinous and granular layers and enter the stratum corneum where they stop proliferating and terminally differentiate. Then, keratinization occurs (98). EGFR inhibitors reduce the expression of the proliferation marker Ki67 suggesting keratinocyte growth arrest and premature differentiation, which ultimately results in abnormal formation and thinning of the stratum corneum (the outermost layer of the epidermis) (99, 100). Moreover, when the EGFR signaling pathway is inhibited, patients become susceptible to pathogenic bacteria, such as *Staphylococcus aureus*. The aggravation of inflammation further inhibits epidermal differentiation and exacerbates keratinocyte damage, leading to the occurrence of eczema-like skin reactions (86). An EGFR knockout model demonstrated that the skin of the mouse became dry and fragile (101). Therefore, EGFR inhibitors damage the natural moisturizing function of the skin and destroy skin homeostasis by damaging the physical barrier of the stratum corneum, leading to dry skin, itching, and rashes.

### Damage of Hair Follicles

EGFR is also expressed abundantly in undifferentiated keratinocytes proliferating in the external root sheath of hair follicles (102). Treatment with EGFR inhibitors induces secretion of pro-inflammatory factors and lymphocyte infiltration, which leads to folliculitis and hair follicle rupture as the disease progresses (85, 103). Folliculitis is also known as acneiform rash and papulopustular exanthema, and the primary lesions are inflammatory follicular papules and pustules. The histopathology of papulopustular exanthema demonstrates purulent folliculitis with ectatic follicular infundibula and rupture of the epithelial lining. Keratin plugs and microorganisms are seen in the dilated infundibulum (104, 105). EGFR plays an essential role during the hair growth cycle (106). In addition, *in vitro* studies have shown that the concentration of EGF regulates the conversion between hair follicle growth and inhibition (107). Some studies have also confirmed that EGFR inhibitors have different effects on hair on different parts of the body. Hair will become brittle, thin, curly, or even be lost, while eyelashes will grow and curl (105, 108, 109).

### Destruction of Skin Homeostasis-Inflammation and Host Immune Activation

In human skin, keratinocytes differentiate to provide a physical barrier in the stratum corneum, but they will also secrete various cytokines, chemokines, and antimicrobial peptides to participate in the innate immune response to resist pathogen invasion (110). Park et al. (111) found that the expression of β-defensin, an antimicrobial peptide produced by human symbiotic bacteria, decreased after using EGFR inhibitors leading to bacterial susceptibility. This may be one of the reasons for skin toxicity.

EGFR inhibitors also activate nuclear factor-κB in both cancer and normal cells, leading to destruction of immune balance and an inflammatory microenvironment (21). When EGFR signaling was inhibited, CCL2, CCL5, and CXCL10 expression levels increased and CXCL8 expression level decreased, which increased leukocyte recruitment and inflammatory infiltration (112). Wan et al. (113) induced a skin rash in female Brown Norway rats with gefitinib and found that macrophages infiltrated to the skin and secreted large amounts of inflammatory cytokines such as TREM-1, CINC-2, and CINC-3.

Furthermore, when EGFR inhibitors are used, the expression level of proapoptotic genes (such as secreted frizzled related protein 1, the apoptosis inhibitor survivin, and BCL2 associated athanogene) are upregulated, and the expression level of antiapoptotic genes (such as death associated protein kinase-1 and apoptosis response zinc finger protein requiem) are downregulated (21). The combination of tumor-induced inflammation with iatrogenic apoptotic lysis may be an important factor of associated skin toxicity.

Severe disruption of skin homeostasis induced by microbial susceptibility, inflammatory activation, and increased apoptosis ultimately leads to the generation of cutaneous toxicity.

### Radiotherapy

Radiation therapy is often combined with chemotherapy or targeted therapy during tumor therapy, and the duration, dose, and area of radiation have a significant impact on the severity of skin toxicity induced by EGFR inhibitors (114). EGFR inhibitors are associated with an increased risk of severe radiation dermatitis during the first few weeks of radiation therapy when radiation damages epidermal basal cells (115). Radiotherapy and chemotherapy cause the release of chemoradiation associated molecular patterns. They play an integral role in the generation of inflammation, which causes adverse skin reactions from EGFR inhibitors more severe and complex (116). In addition, skin xerosis caused by cetuximab may aggravate dermatitis caused by radiotherapy (117).

## Factors Leading to Fatal Skin Toxicity

Severe and fatal skin toxicities occur in only a small number of patients treated with EGFR inhibitors, but the pathogenic mechanism remains unclear. Le-Rademacher et al. (118) found that androgens may mediate adverse skin reactions caused by EGFR inhibitors, and anti-androgen therapy may be a method to treat or alleviate skin toxicity. Another study showed that patients with high sebaceous gland activity and sebum secretion were more sensitive to EGFR inhibitors and developed acneiform rash more frequently (119). Takahashi et al. (120) also found that men and high-weight patients who used EGFR inhibitors were more susceptible to severe skin toxicity. High male hormones, high sebum secretion, and smoking (more prevalent in males than females) are risk factors causing male lung cancer patients to have more severe adverse skin reactions.

Other risk factors leading to skin toxicity after EGFR-TKI treatment require further investigation. For example, individuals with mutations in interleukin-36 receptor antagonist may be at an increased risk of AGEP development after drug treatment (89). Ethnicity may also be a risk factor. The frequency of EGFR-TKI-associated SJS/TEN is higher in Asian countries than in other regions (121). In addition, sulfonamides, anti-epileptic drugs (carbamazepine, phenytoin, phenobarbital, lamotrigine), nonsteroidal anti-inflammatory drugs of the oxicam type, and allopurinol have been shown to be high-risk drugs for inducing delayed type hypersensitivity SJS/TEN. Therefore, the possible risk of serious adverse effects should be considered when EGFR inhibitors are used in combination with these drugs (122). When more than one susceptibility factor for lethal skin toxicity exists, the treatment of related cancers with EGFR inhibitors should be evaluated early and continuously monitored. The treatment of skin toxicity should be started early to reduce the pain and death risk of patients.

## The Relationship Between Skin Toxicity and Anticancer Efficacy

EGFR is essential for maintaining the development and normal physiological functions of the epidermis in the skin. The main cause of skin toxicity is the targeting effect of anti-tumor drugs on wild-type EGFR. It has been suggested that skin response can be used as a biomarker of EGFR efficacy (123). A review of 116 patients treated with cetuximab and panitumumab by Jaka et al. (124) also confirmed that more severe rashes were associated with better outcomes. Because of the observed positive correlation of rash with efficacy, studies have suggested a new administration in which the dose is increased until the rash is most tolerable to the patient. The severity of EGFR inhibitor-induced skin toxicity is positively correlated with the therapeutic effect, making related skin toxicity a potential marker for predicting drug efficacy.

Determining how to predict whether a patient will have skin toxicity is an important area of investigation In 2004, Amador et al. (125) found that the number of single sequence repeats in EGFR intron 1 was related to the skin toxicity and anti-tumor activity of EGFR inhibitors. Kimura et al. (126) found that compared with patients who did not show any skin toxicity, the plasma macrophage inflammatory protein level was significantly decreased in patients with skin toxicity, suggesting that macrophage inflammatory protein levels in plasma might be a predictor of dermal toxicity in patients treated with gefitinib. In 2012, Moreno Garcia et al. (127) observed that elevated plasma creatine kinase was associated with EGFR-TKI-induced rash, and *in vitro* experiments showed that the expression level of cytosolic isoforms of creatine kinase-brain increased after EGFR-TKIs stimulated human keratinocytes. Steffens et al. (128) found that patients treated with higher erlotinib/O-demethyl-erlotinib (O-demethyl-erlotinib is the main active metabolite of erlotinib) had longer progression free survival and overall survival. They were also more prone to adverse skin reactions. The occurrence of rash was positively correlated with progression free survival and overall survival. The identification of biomarkers for severe skin toxicity can help doctors to take preventive measures to prevent severe or even fatal skin toxicity in patients. These blood biomarkers can predict drug efficacy or serious skin toxicity earlier than the occurrence of a skin rash, and it is more appropriate to predict the effect of EGFR inhibitors for patients who are not prone to skin toxicity.

Whether the efficacy of all EGFR inhibitors can be measured by skin toxicity is debatable. When applying the less targetable first-generation EGFR-TKIs (e.g., erlotinib, gefitinib, afatinib), the targeted toxicity of the skin may serve as a biomarker to measure anticancer efficacy. However, skin toxicity as an indicator of efficacy is not applicable to all EGFR-TKIs. Osimertinib (third-generation EGFR-TKIs) is typically used for treatment of NSCLC patients with the T790M resistance mutation. It has significantly greater activity against tumor EGFR with mutations del19, L858R, and T790M than wild-type EGFR (129, 130). The incidence of adverse skin reactions is lower with osimertinib than with erlotinib, but it is an effective treatment for NSCLC with T790M mutations. Further investigations are needed because the use of skin toxicity as an indicator of EGFR inhibitor efficacy is incomplete (131).

## Therapeutic Strategies for Skin Toxicity

Up to 90% of cancer patients treated with EGFR inhibitors have skin adverse reactions. Of these, 76% of patients reported that they interrupted the EGFR inhibitor therapy, 32% of patients completely discontinued the EGFR inhibitor therapy, and 60% of patients reduced the dose of the EGFR inhibitor (132, 133). The most common skin toxicity caused by EGFR inhibitors is follicular papulopustular exanthemas, also known as follicular rash. It usually occurs on the head, back, and upper chest in the first few weeks of treatment. The lesions disappear without sequelae upon withdrawal of the EGFR inhibitor (134). Sebostasis, epidermal atrophy, itchy eczema, skin xerosis, paronychia, and changes in hair (such as hair and eyelashes) often occur after 1 to 2 months of treatment (132). In addition, skin toxicity caused by EGFR inhibitors is often accompanied by severe pain and extreme itching causing patients to endure physical pain and psychological stress. The clinical manifestations and basic grades of common skin toxicities (e.g., papulopustular exanthemas, pruritus, xerosis, paronychia, hair changes) caused by EGFR inhibitors are shown in **Table 3**.

**Table 3:** Clinical manifestations and classification of common skin toxicities of EGFR inhibitors.

Common skin toxicities	Clinical manifestations	Grades criteria (NCI-CTCAE v 5.0) (135)
Papulopustular exanthemas	Predominantly occurring on the face back and upper chest within two weeks from the start of EGFR inhibitor treatment; manifests as red papules and/or pustules without comedone (136, 137)	Grade 1: Papules and/or pustules covering <10% BSA, with or without pruritus or tenderness Grade 2: Papules and/or pustules covering 10-30% BSA, with or without symptoms of pruritus or tenderness; with psychosocial impact; limiting instrumental activities of daily living; papules and/or pustules covering >30% BSA but mild symptoms Grade 3: Papules and/or pustules covering >30% BSA, with moderate to severe symptoms; limiting self-care activities of daily living; associated with local superinfection with oral antibiotics indicated Grade 4: Papules and/or pustules covering any % BSA; with unlimited symptoms; associated with extensive superinfection with IV antibiotics indicated; life-threatening consequences Grade 5: Death
Pruritus	A disorder characterized by an intense itching sensation, accompanies the papulopustular exanthemas and xerosis at onset (138)	Grade 1:Mild or localized; topical intervention indicated Grade 2: Intense or widespread; intermittent; skin changes from scratching (e.g., edema, papulation, excoriations, lichenification, oozing/crusts); limiting instrumental activities of daily living Grade 3: Intense or widespread; constant; limiting self-care activities of daily living or sleep; oral corticosteroid or immunosuppressive therapy indicated
Skin Xerosis	Dry skin, often accompanied by pruritus, scaly, flaking skin appears over the extremities, the fingertips and toes may develop dry areas with cracks, or fissures (85, 139)	Grade 1:Covering <10% BSA and no associated erythema or pruritus Grade 2: Covering 10-30% BSA and associated with erythema or pruritus; limiting instrumental activities of daily living Grade 3: Covering >30% BSA and associated with pruritus; limiting self-care activities of daily living
Paronychia	Nail-fold edema or erythema, damaged skin around nail, disruption of the cuticle, nail-plate separation, granulation tissue formation (140)	Grade 1: Nail fold edema or erythema; disruption of the cuticle. Grade 2: Nail fold edema or erythema with pain; associated with discharge or nail plate separation; limits instrumental activities of daily living; topical or oral anti-infective therapy indicated Grade 3: Surgical intervention or intravenous antibiotic treatment indicated; limits self-care activities of daily living
Hair changes	Hair become brittle, thin, curly, or even be lost, eyelashes grow and curl (85, 109)	/

Grading of papulopustular exanthemas pruritus, xerosis, and paronychia according to the NCI-CTCAE version 5.0. CTCAE, Common Terminology Criteria for Adverse Events; NCI, National Cancer Institute; BSA, body surface area.

Treatment strategies for skin toxicity caused by EGFR inhibitors currently include empirical treatment and expert consensus in countries such as the United Kingdom (141), Germany (142), Taiwan (136), France (143, 144), Italy (145), and Spain (146). These consensuses general principles of treatment about skin toxicities are consistent but differ slightly. They mainly describe treatment strategies for common skin toxicities caused by EGFR inhibitors, but not for fatal skin toxicities. Moreover, most treatments are focused on alleviating symptoms without effective etiological treatment. There is no recognized authoritative guide for the treatment of EGFR inhibitors related skin toxicity. This is an urgent clinical problem that needs to be solved.

### Symptomatic Treatment

#### Papulopustular Exanthemas

When grade 1 rash occurs, the patient can continue to use EGFR inhibitors and to use non-alcoholic emollients (141). Reduction or discontinuation of EGFR inhibitors should be considered when grade 2 rash duration is unmanageable or the patient is unable to tolerate it. EGFR inhibitor therapy should be temporarily discontinued when ≥ grade 3 rash appears (141). Therapeutic measures are shown in more detail in **Table 4**.

**Table 4:** Treatments of papulopustular exanthemas caused by EGFR inhibitors.

Grade	Therapeutic measures
1	Continue EGFR inhibitors at the original dose; moisturizing and sunscreen (sun protection factor SPF ≥30); topical antibiotics (clindamycin 1-2% gel, erythromycin 1%, nadifloxacin 1%; fusidic acid 2% or preparations containing metronidazole 0.75%); topical calcineurin inhibitors (tacrolimus 0.1% ointment or pimecrolimus 1% cream bid); reassess after at least 2 weeks or any worsening of symptoms (136, 141, 142, 147)
2	Symptom deterioration or patient intolerance (reduction or discontinuation of EGFR inhibitors); moisturizing and sunscreen; topical corticosteroids (hydrocortisone 1-2.5%, prednicarbate 0.02% cream, mometasone furoate 0.1%, desoximetasone 0.25%); topical antibiotics (clindamycin 1-2% gel, erythromycin 1%, nadifloxacin 1%; fusidic acid 2% or preparations containing metronidazole 0.75%); topical calcineurin inhibitors (tacrolimus 0.1% ointment or pimecrolimus 1% cream bid.); Oral antibiotics [such as tetracycline (250-500 mg), doxycycline (100-200 mg, bid), oxytetracycline (500 mg, bid) or minocycline (100 mg, bid)]; antihistamines; reassess after at least 2 weeks or any worsening of symptom (133, 136, 141, 142, 147–149)
3	Temporary discontinuation of EGFR inhibitors; moisturizing and sunscreen; oral antibiotics [such as tetracycline (250-500 mg), doxycycline (100-200 mg, bid), oxytetracycline (500 mg, bid) or minocycline (100 mg, bid)] plus a short course of oral corticosteroid (prednisolone 0.5-1 mg/kg/day for 5–7 days); consider oral isotretinoin at low doses (20-30 mg/day); reassess after at least 2 weeks or any worsening of symptoms (133, 141, 142, 146, 147, 149)
4	Same as grade 3
5	Discontinuation of EGFR inhibitors

#### Pruritus Due to Papulopustular Exanthemas and Xerosis

Grade 1-2 can be treated with topical steroids (0.05% clobetasol), and oral antihistamines (cetirizine, loratadine, etc.) can be used for grade 3 pruritus. In addition to the drugs mentioned above, gamma-aminobutyric acid agonists, neurokinin-1 receptor antagonists, antidepressants, corticosteroids, and other drugs can be added for treatment (150, 151). However, caution should be taken to avoid systemic steroids as they can have acneiform rash-like side effects (152). However, when a rash of grade ≥ 3 occurs, systemic dexamethasone or prednisolone is usually used for treatment (83).

Once bacterial infection occurs, systemic antibiotics can be selected for treatment based on a drug sensitivity test (153). If local use of metronidazole is not enough to control symptoms of papulopustular lesions, then they can be treated by oral tetracycline (152, 154). For skin toxicity with pustules and a large amount of exudate (typically grade 3 or higher), the use of both tetracycline and saline compresses (15 minutes, 2 to 3 times a day) can effectively control inflammation (152, 155). Doxycycline is recommended for patients with renal insufficiency, and minocycline is recommended for patients living in areas with high ultraviolet exposure (156). At the same time, attention should be paid to the intestinal microflora disorder caused by long-term systemic antibiotics in the treatment of rash (157).

In addition to the conventional treatment mentioned above, Bavetta et al. (158) found a significant improvement in skin symptoms after 4 weeks of treatment with a cream containing 1.5% polydatin (a natural precursor of resveratrol), suggesting that it may be used as an adjunctive agent for prophylactic treatment of papulopustular exanthemas and as an alternative to corticosteroids. Lacouture et al. (159) showed that although BRAF inhibitors can activate mitogen-activated protein kinase downstream of EGFR, topical use of BRAF inhibitor LUT014 can improve the skin toxicity induced by EGFR inhibitors cetuximab or panitumumab. Additionally, topical use of recombinant human EGF may ameliorate the rash produced by EGFR inhibitors by regulating the normal proliferation and differentiation of keratinocytes and reducing the expression of inflammatory factors (93).

#### Skin Xerosis

Patients with dry skin should use moisturizing emollients several times a day, avoid bathing with soap and hot water, and use emollients to moisturize the skin after cleansing (147). Water-based creams aggravate dry skin and very greasy emollients increase the risk of folliculitis. Therefore, ointment is recommended for the care of dry skin. Specific emollients and soap substitutes are recommended by the United Kingdom EGFR-TKI expert consensus on adverse event management published in 2015 (141). In addition, skin dryness with eczematous lesions is treated with topical steroids (160).

#### Paronychia

Paronychia can be extremely painful to the patient, leading to difficulty in walking and limited mobility by affecting the nails of the fingers and toes (148). A retrospective study by Osio et al. (103) found that patients using EGFR inhibitors for more than 6 months had a > 50% chance of developing paronychia. Patients with paronychia can be treated with silver nitrate, preservatives, topical corticosteroids, and antibiotics. For grade 1 and 2 paronychia, topical betamethasone valerate (2-3 times, qd) is recommended; for grade 3 paronychia, local use of clobetasol cream (2-3 times, qd) is recommended. Patients with periungual granulomas can be treated with nitrate first. If there is no response, then curettage and cauterization can be utilized (133, 148, 161). Therapeutic measures are shown in more detail in **Table 5**.

**Table 5:** Treatments of paronychia caused by EGFR inhibitors.

Grade	Therapeutic measures
1	Continue EGFR inhibitors at original dose; antiseptic hand bath (povidone iodine 1:10, potassium permanganate 1:10000, white vinegar in water 1:1); topical betamethasone valerate (2-3 times, qd); reassess after 2 weeks (133, 142)
2	Continue EGFR inhibitors at original dose; silver nitrate solution 20% weekly (administer cryotherapy or other chemical/electric cauterization if granulation); povidone-iodine 2% ointment; topical betamethasone valerate 0.1% ointment (2-3 times, qd); oral antibiotics are recommended; reassess after 2 weeks (133, 136, 142, 149)
3	Temporary discontinuation of EGFR inhibitors; topical clobetasol cream (2-3 times, qd); povidone-iodine 2% ointment; systemic antibiotics oral or intravenously following pathogenic culture; continue to apply topical antibiotics; reassess after 2 weeks (136, 142, 148, 149, 161)

#### Hair Changes

Topical minoxidil is recommended for non-cicatricial alopecia of the head caused by EGFR inhibitors. Topical steroids are recommended for inflammatory and cicatricial alopecia (160). Curled hypertrophic long eyelashes can be trimmed, and facial hirsutism can be treated with laser hair removal (160).

### Management of Lethal Severe Cutaneous Adverse Reactions

Rare but fatal adverse skin reactions such as AGEP, SJS, TEN, and SJS/TEN may be caused when EGFR inhibitors are used. Their clinical manifestations and related therapeutic measures are shown in the **Table 6**. The general treatment principle is to stop the relevant EGFR inhibitors, reduce fluid loss, replenish body fluids, control pain, and provide adequate nutrition (88). AGEP symptoms usually resolve rapidly after discontinuing EGFR inhibitors. Topical corticosteroids and systemic antihistamines are recommended for symptom control (168).

**Table 6:** Clinical manifestations and therapeutic measures of severely fatal skin toxicities.

Lethal Skin Toxicities	Clinical manifestations	Therapeutic measures
AGEP	Fever ≥38°C, sterile non-follicular pustules on the base of the erythema, leukocytosis, neutrophils ≥7000, mild eosinophilia, multiple organs involved (89, 162)	EGFR inhibitors withdrawal; topical corticosteroids, systemic antihistamines (162, 163)
TEN	Fever≥38°C, influenza-like syndrome, respiratory tract symptoms, Lymphopenia, transitory neutropenia, mild cytolysis, blisters, multiple organs involved, Nikolsky’s sign, skin detachment ≥30% (162)	EGFR inhibitors withdrawal; corticosteroids, cyclosporine, intravenous immunoglobulins, TNF-α inhibitors; plasmapheresis (163, 164)
SJS	The clinical manifestations were similar to TEN, skin detachment <10% (162)	SJS treatment strategy is the same as TEN
NME	Annular-circinate, erythematous, scaly rash, superficial epidermal necrosis, plasma glucagon levels increased, diabetes mellitus or glucose intolerance (11, 165)	EGFR inhibitors withdrawal; oral prednisolone (0.5 mg/kg/d); octreotide and lanreotide; clobetasol propionate ointment 0.05% (28, 165, 166)
DIHS	Fever ≥38°C, extensive rash, atypical lymphocytosis, eosinophilia, lymphadenopathy, multiple organ dysfunction, reactivation of human herpes virus 6 and human herpes virus 7 (35, 41)	EGFR inhibitors withdrawal; oral prednisolone (0.5 mg/kg/day); cyclosporine (5 mg/kg/day) (41, 167)

For more severe skin toxicities such as SJS, TEN, SJS/TEN, conservative treatment (e.g., applying emollients) recommends maintaining skin integrity and preventing fluid loss. Surgical debridement is recommended only when infection occurs. Aggressive treatment recommends removing exfoliated epidermis that may be infected (88). In subsequent treatment, emollients and steroid creams can be used alternately for moisturizing and anti-inflammation. Gauze soaked with betadine can be used for bandaging (169).

A meta-analysis of the literature suggested that cyclosporine was effective in reducing mortality from SJS/TEN, and the combination of cyclosporine and systemic steroids may be an effective treatment for SJS/TEN (170–172). Mucosal damage caused by TEN often affects the eyes, gastrointestinal tract, and respiratory tract. TEN often causes eye keratitis and corneal erosion. It is recommended to consult an ophthalmologist, use antibiotic eye drops to prevent bacterial infection, and use eye lubricant combined with topical corticosteroids for the treatment of eye complications. Attention should be paid to secondary glaucoma caused by steroid treatment (173, 174). Oral ulcers are the most common in TEN and can be treated with topical lidocaine gel or cocaine mouthwash. In addition, the mucous membrane of the respiratory tract may fall off and cause respiratory distress that requires management by a specialized physician (169).

### Preventive Measures

Due to the important role of EGFR in the normal physiological function of the skin, the incidence of adverse skin reactions caused by EGFR inhibitors is 60%-85%. These adverse reactions often lead to the reduction or even withdrawal of antitumor drugs (175). Therefore, preventing skin toxicity is increasingly gaining attention by investigators. Prophylactic use of emollients, sunscreen, mild body wash and facial cleansers, are ointment containing EGF are beneficial measures to prevent or reduce skin toxicity in patients treated with EGFR inhibitors (141, 142, 176).

A phase III clinical trial conducted in Canada in 2016 showed that the preventive use of minocycline (100 mg twice a day for 1 month) before erlotinib did not reduce the incidence of rashes but reduced the incidence of grade 3 skin toxicity while not affecting efficacy (177). Takahashi et al. (120) also showed that the grade of acneiform rash was lower after preventive use of minocycline. Meanwhile, Ichiki et al. (178) found that prophylactic use of minocycline (50 mg twice a day for 4 weeks) reduced rashes and paronychia induced by afatinib. In addition, preventive use of minocycline and topical corticosteroids may be effective for afatinib-induced paronychia, but elevated transaminase was found in patients during the use of minocycline. Therefore, long-term use of minocycline should be noted for possible liver damage (179).

Preventive use of doxycycline (100 mg twice a day for 4 weeks) can reduce the incidence of grade 2 or high adverse skin reactions caused by dacomitinib (180). In the treatment of refractory metastatic colorectal cancer with panitumumab, prophylactic doxycycline (100 mg twice a day for 6 weeks) and topical moisturizers, sunblock, and 1% hydrocortisone cream reduced the incidence of panitumumab-induced skin toxicity higher than grade 2 by 50% (181). Petrelli et al. (182) conducted a systematic review and meta-analysis of studies on the use of antibiotics to prevent skin rashes before 2016 and found that preventive use of minocycline or doxycycline reduced the absolute risk of all skin rashes (grade1-4) and severe skin rashes (grade 2-4) by 10% and 25%, respectively.

A randomized, open-label trial confirmed that tetracycline (250 mg twice a day for 4 weeks) was effective for afatinib-induced acneiform rash, and prophylactic use of tetracycline reduced the incidence and severity of rashes (183). However, Jatoi et al. (184) found that prophylactic use of tetracycline (500 mg orally twice a day for 28 days) did not reduce the incidence or severity of rashes induced by EGFR inhibitors. The different doses may be the reason for the inconsistent research results. Petrelli et al. (182) concluded that tetracycline could significantly reduce the incidence of severe rash induced by EGFR inhibitors after analyzing 13 clinical studies. Hofheinz et al. (185) recommended prophylactic use of antibiotics (such as tetracycline, doxycycline, and minocycline) on the first day of EGFR therapy to reduce the severity of adverse skin reactions and improve patient compliance. However, Italian experts do not recommend the preventive use of antibiotics as a treatment method to prevent serious skin toxicity of EGFR inhibitors (145). For skin toxicity caused by EGFR inhibitors, whether to use antibiotics prophylactically needs to be determined by comprehensively considering the situation of the patient.

In addition to the aforementioned studies on the preventive use of antibiotics, nonsteroidal anti-inflammatory drugs may also play a role in preventing EGFR inhibitor-related rashes (186). Local prophylactic use of 3% chloramphenicol + 0.5% prednisolone ointment significantly reduced the severity of facial papulopustular exanthemas induced by EGFR inhibitors (175). Although studies have shown that preventive use of vitamin K3 cream does not reduce the number of papulopustular exanthemas (187), preventive use of vitamin K1 cream can reduce the incidence of grade 2 or higher rashes (188). A randomized single-blind trial conducted by Chayahara et al. (189) showed that compared with the control group adapalene treatment did not prevent acneiform rash and may have harmful effects. Therefore, adapalene is not recommended to prevent acneiform rash caused by EGFR inhibitors.

## Conclusion

To avoid the lethal skin toxicity caused by EGFR inhibitors, more targeted drugs need to be developed as well as conducting further investigations on efficacious preventive measures before cancer treatment and beneficial treatment measures after adverse skin reactions occur. In addition, there is no official or unified guidelines to deal with skin toxicities induced by EGFR inhibitors. Therefore, to create an authoritative guide would be beneficial to clinicians. The occurrence of adverse skin reactions and fatal skin toxicity are the most widespread reasons that limit anti-tumor treatments with EGFR inhibitors. Using the existing evidence for prevention and treatment should be an area of interest for medical staff and scientific researchers.

This paper summarized the cellular and molecular mechanisms of EGFR signaling and adverse skin reactions caused by EGFR inhibitors to provide ideas for the use of EGFR inhibitors and the prevention of related skin toxicity in cancer treatment. Effectively preventing and treating skin toxicity without damaging the anti-tumor efficacy of EGFR inhibitors is the ultimate goal we want to achieve. Treatment after the occurrence of skin toxicity is the key to effective anti-tumor treatment and a good prognosis of patients. This will require medical care providers to summarize and record more treatment details during their daily work, formulate a series of effective treatment schemes, and publish these results.

## Author Contributions

LY conceived and supervised the project. LYP summed up the literature and drafted the manuscript. FRQ collected and organized the inhibitors and revised the manuscript. All authors contributed to the article and approved the submitted version.

## Funding

This study was funded by the Chongqing Clinical Pharmacy Key Specialties Construction Project.

## Conflict of Interest

The authors declare that the research was conducted in the absence of any commercial or financial relationships that could be construed as a potential conflict of interest.

## Publisher’s Note

All claims expressed in this article are solely those of the authors and do not necessarily represent those of their affiliated organizations, or those of the publisher, the editors and the reviewers. Any product that may be evaluated in this article, or claim that may be made by its manufacturer, is not guaranteed or endorsed by the publisher.
